# Silent Springs: Why Are All the Frogs “Croaking”?

**DOI:** 10.1371/journal.pbio.1000198

**Published:** 2009-09-15

**Authors:** Matthew C. Fisher

**Affiliations:** Department of Infectious Disease Epidemiology, Faculty of Medicine, Imperial College London, London, United Kingdom

## Abstract

Amphibians are a fabulously successful group of animals; however, it is increasingly clear that they are experiencing extinction rates that far exceed those experienced by other classes of vertebrates. A new book examines the various reasons why amphibians are so threatened, and what can be done about it.

**Figure pbio-1000198-g001:**
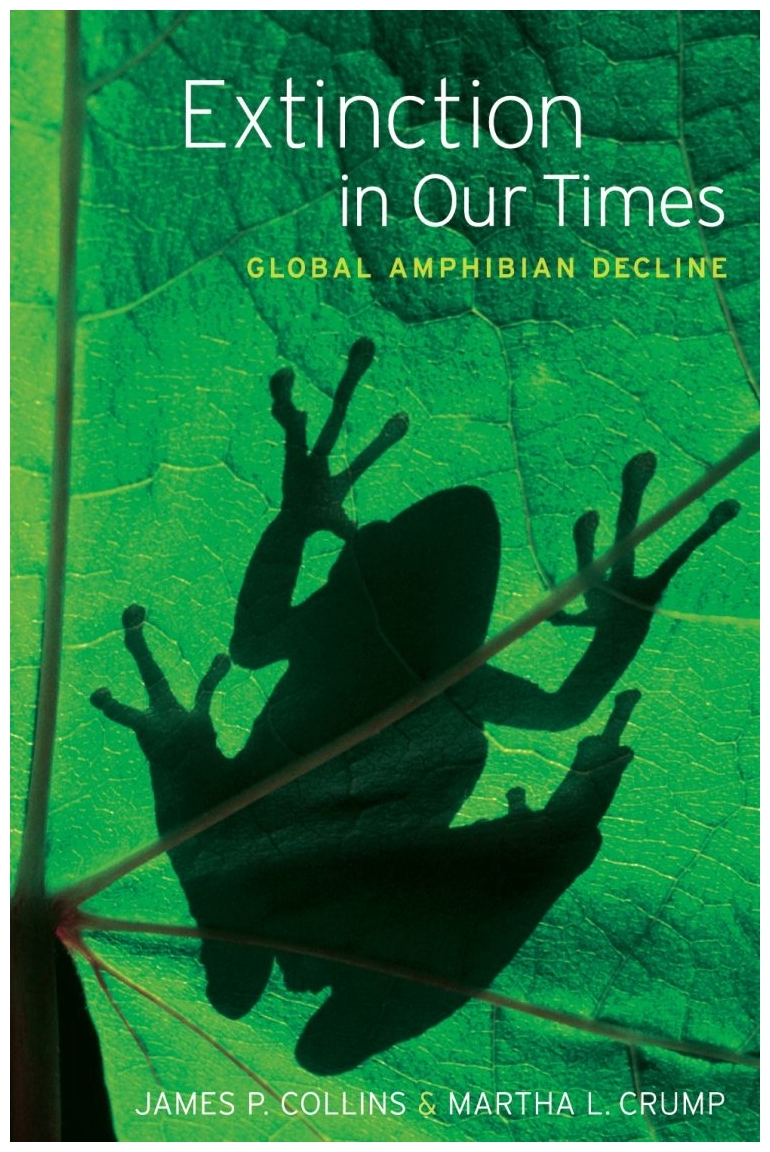
Collins JP, Crump ML (2009) Extinction in our times: global amphibian decline. New York: Oxford University Press USA. 304 p. ISBN (hardcover): 978-0-195-31694-0 US$29.95.

[Fig pbio-1000198-g001]Amphibians became the most ancient class of land-dwelling vertebrates when, 360 million years ago, primitive amphibia such as *Ichthyostega* first hauled themselves out of their aquatic environment. Since that ancient event, the amphibia have ramified into over 6,300 species of breathtaking diversity: they inhabit all continents (excepting Antarctica), from deserts to altitudes of over 5,300 metres, manufacture an astoundingly complex arsenal of chemicals, and undergo complex metamorphic transitions that bridge aquatic and terrestrial ecosystems.

Scientists have long been captivated by the success and rich diversity of amphibians, but over the past 30 years, their wonder has turned to horror. Jaime Bosch's experience was typical. During the 1990s, the Spanish researcher used to climb the Sierra de Guadarrama each year to study the mating choruses of midwife toads for his PhD. This species of amphibian is unusual in that the males care for fertilised eggs entwined around their hind-legs and use a simple, monotonic call to locate the opposite sex; they were numerous in this pristine montane environment, and finding adequate sample-sizes was never a problem. But in the summers of 1997 and 1998, Bosch noticed that something in his study populations was seriously awry; although tadpoles were successfully hatching and developing, upon metamorphosis, the toads took ill and died by the thousands. In the span of a few years, the midwife toads became all but extinct in the Sierras [1].

This simple story has been recapitulated now dozens of times. During the 1970s and 1980s, naturalists and scientists who study amphibians first started to note that their study species were becoming scarcer, sometimes disappearing right in front of their eyes. As modern communications developed, these increasingly networked researchers began to realise that their separate stories were not simply unique anecdotes, but part of a widespread phenomenon: something was happening to amphibians worldwide.

*Extinction in Our Times: Global Amphibian Decline* is a 304-page book comprising ten chapters that set out the key events that led to a realisation that amphibian declines were not only real, but were also occurring globally. The authors, James P. Collins and Martha L. Crump, are key leaders in this field who have long been researching amphibian declines. The book picks up at the First World Congress of Herpetology, held in Canterbury in 1989. It was here that researchers began to piece their separate stories together to reveal a unifying theme: Amphibian species were going extinct at rates that far exceeded background extinction, and were above those seen in other classes of vertebrates. Although a broad consensus was reached that these global amphibian declines were real, their proximate drivers were entirely unclear and often hotly debated. *Extinction in Our Times* sets out to document this complex field of research in which competing scientific agendas, industrial interests, governmental policy, and conservation ethics intersect in a (sometimes conflicting) effort to identify, and then mitigate, amphibian declines.

Collins and Crump have written an impartial and detailed overview of the known, and unknown, factors that are driving amphibian declines. It is now clear that the accelerating crash in global amphibian diversity is unlike that described in other classes of vertebrates. This is perhaps because, uniquely, amphibians are unusually susceptible to an additional driver, infectious disease. Principally, the pathogenic fungus *Batrachochytrium dendrobatidis* (*Bd*) has taken center stage in the research agenda because it not only infects an unusually wide range of amphibian species, over 387 at the last count [2], but also appears to be spreading into uninfected populations at alarming rates. For instance, the local extinction in the Sierra de Guadarrama that Bosch witnessed was caused by the emergence of chytridiomycosis in his population of midwife toads, and waves of spread have been documented in the Americas and Australia. However, as Collins and Crump show, it is incorrect to overstate the importance of *Bd* and chytridiomycosis in causing declines and extinction events. It is increasingly being recognised that the principal cause underlying the loss of amphibian species is the attrition of their natural environments via a plethora of anthropogenic changes; these include the introduction of nonnative species, overharvesting for commerce, land use change and environmental pollutants. For instance, as has been elegantly shown by Rohr et al. [3], agrochemicals exacerbate disease emergence in northern leopard frogs via several causal mechanisms. Their study makes an important point: there are many drivers of global disease declines, and these may be synergistic, or even antagonistic. Collins and Crump recognise this and are at pains to present a balanced analysis of these various scenarios, with separate chapters and sections dealing with the multiplicity of potential drivers. Correctly, the authors steer clear of future gazing about the potential effects of changing climates on amphibian populations. Although there are good reasons to believe that global warming is involved in amphibian declines, perhaps by exacerbating the effects of *Bd* on amphibian populations [4],[5], the necessary evidence to prove or disprove this hypothesis is still lacking [6],[7]; *Extinction in Our Times* avoids the use of hyperbole and overstatement, and this is refreshing to find in a field where dialogue can have a tendency to become overheated.

Collins and Crump adopt an informal, yet authoritative style and their dialogue is peppered with anecdotes that lead to a genuine sense of excitement about the race, the collaborations and the competitions that led to the identification of the causes of amphibian declines, as well as the existing uncertainties. Critically speaking, their use of infectious disease terminology is a little loose (for instance, we normally talk of ”transmission„ rather than ”selection„ to describe the process of infection between hosts); however, this does not undermine the value of this book as an excellent synthesis of the field. Finally, and importantly, the authors contextualise the science within the policy and ethical frameworks that will determine whether we are able to do anything to slow down this increasingly rapid leakage of biodiversity. Currently, there is a tension between conservationists who propose to embed amphibian diversity in “Amphibian Arks” to ride out the storm, and others who argue that this is simply putting “canaries under intensive care.” To an extent, both camps are right, and a balanced, context-dependent risk analysis needs to be undertaken for the many at-risk species in order to determine the best course of action. However, as Collins and Crump state, “We intend our analysis to be a roadmap and hope that future research can progress even faster as it builds upon a clearer vision of what we already know.” Others go further and suggest that we have already run out of time and are simply “rearranging deckchairs on the Titanic.” Whoever is correct, *Extinction in Our Times* is a valuable and well-considered addition to the arsenal of evidence that we need to execute a rapid response to this accelerating catastrophe.

Kermit the Frog's famous song, “It's Not Easy Bein' Green,” has never been more apt—or poignant.
